# Effect comparison of neuroendoscopic vs. craniotomy in the treatment of adult intracranial arachnoid cyst

**DOI:** 10.3389/fsurg.2022.1054416

**Published:** 2023-01-06

**Authors:** Jianfeng Liang, Kai Li, Bin Luo, Jun Zhang, Peng Zhao, Changyu Lu

**Affiliations:** ^1^ Department of Neurosurgery, Peking University International Hospital, Beijing, China; ^2^ Department of Neurosurgery, Beijing Tiantan Hospital, Capital Medical University, Beijing, China

**Keywords:** neuroendoscopic, craniotomy, intracranial arachnoid cyst, surgery, complication

## Abstract

**Purpose:**

Intracranial arachnoid cysts are common, accounting for about 1%–2% of intracranial space-occupying lesions. There is controversy over the method of surgical intervention, and in order to provide guidance for surgical decision making, this study compares the efficacy of craniotomy vs. neuroendoscopic surgery in treating arachnoid cysts.

**Methods:**

The adult patients with arachnoid cyst admitted to our department from October 2016 to August 2021 were retrospectively analyzed. Thirteen adult patients were recruited, and divided into two groups: neuroendoscopic group (group A) and craniotomy group (group B). We compared the gender, age, clinical symptoms, preoperative and postoperative cyst sizes, symptom improvement, complications, length of hospital stay, and hospital costs between two groups to analyze the therapeutic effects of these two surgical methods.

**Results:**

The cost of hospitalization in group A was significantly lower than that in group B (47,292.8 vs. 65,151.8 yuan, *P* < 0.05), and there was no difference in the length of hospital stay between the two groups. The preoperative cysts in group A were significantly larger than those in group B (6.38 vs. 2.97 cm, *P* < 0.05). In groups A and B, the short-term symptom improvement rates were 100% and 75.0%, respectively. The long-term symptom improvement rates were 77.78% and 75.0% (*P* > 0.05), respectively.

**Conclusion:**

Both neuroendoscopic and craniotomy have good curative effects for the treatment of intracranial arachnoid cysts. There was no significant difference in the outcomes between the two surgical techniques. The cost of hospitalization can be reduced with neuroendoscopic surgery. Neuroendoscopic treatment is recommended for large intracranial arachnoid cysts, and craniotomy is suitable for small intracranial arachnoid cysts.

## Introduction

Intracranial arachnoid cyst is a benign cyst occurring in the central nervous system, which is closely related to arachnoid. The cystic fluid is often colorless, clear, and similar to cerebrospinal fluid. Intracranial arachnoid cyst can cause neurological symptoms clinically, and the incidence rate of these legions is about 1% of intracranial space-occupying lesions (1).

In recent years, the application of head CT and/or MRI has become widespread with the development of imaging examinations. Usually, an intracranial arachnoid cyst is found by imaging examination after brain trauma (2), and most of the patients have no clinical symptoms (3). Thus, intracranial arachnoid cysts are often found by accident, and the clinical detection rate of intracranial arachnoid cysts is significantly higher than before (2).

Intracranial arachnoid cysts are usually stable; the size mostly remains unchanged, and some cysts might shrink and disappear (4, 5). Compared with pediatric intracranial arachnoid cyst patients, the adult patient's lesion is more stable (6). Thus, there are fewer adult intracranial arachnoid cyst patients with an indication for surgery. The indications for surgery for an intracranial arachnoid cyst include intracranial hypertension, seizure, dysneuria, hemorrhage, and an enlarged cyst (7, 8).

The surgical approaches to intracranial arachnoid cyst include endoscopic surgery, shunt, craniotomy, and drainage (9, 10). Craniotomy and endoscopic surgery are common approaches to intracranial arachnoid cyst, but it is still unclear which approach has better efficacy (10, 11).

In this study, we retrospectively followed up adult patients with intracranial arachnoid cysts who underwent craniotomy or endoscopic surgery in our hospital and compared the improvement of clinical symptoms, complications, treatment costs, and hospitalization days in order to provide a basis for the selection of surgical methods for the clinical treatment of intracranial arachnoid cysts.

## Materials and methods

### General data

The present study is a retrospective study on 13 adult patients with intracranial arachnoid cyst who were admitted to our hospital for medical treatment between October 2016 and August 2021. The patients were divided into two groups depending on the surgical approach. The patients in group A underwent endoscopic surgery (*n* = 9), and the patients in group B underwent craniotomy (*n* = 4). The patient’s general data are listed in Table 1.

**Table 1:** Overview of patients.

No	Group	Sex	Age (years)	Symptoms	Follow-up (months)	MD (cm, pre)	MD (cm, post)	Complication (post)	Location
1	A	Female	26	Dizziness, giddiness, memory deterioration	64	6.29	6.17	Intracranial infection, hyponatremia	Saddle
2	A	Male	36	Headache	62	12.90	12.68	None	Left frontal and temporal lobes
3	A	Female	44	Headache	46	5.54	5.25	Hyponatremia	Prepontine cistern
4	A	Female	60	Dizziness	38	5.85	4.26	LDVT	Right frontal lobe
5	A	Female	63	Dizziness decreased muscle strength in left limb	39	6.12	3.08	None	Right temporal lobe
6	A	Male	36	Headache	26	6.65	5.27	None	Right temporal lobe
7	A	Male	19	Seizure	13	3.76	1.37	Intracranial infection, hyponatremia, hypokalemia	Right frontal lobe
8	A	Female	26	Ataxia, dysarthria,	11	6.10	4.2	None	Left CPA
9	A	Female	33	Dizziness, tinnitus, ataxia	11	4.23	2.94	None	Right CPA
10	B	Male	35	Hand tremor	69	3.69	3.65	None	Left temporal lobe
11	B	Male	35	Dizziness, headache, vomiting	61	4.45	3.58	None	Cerebellum
12	B	Female	66	Headache	38	1.54	1.04	LDVT	Left CPA
13	B	Female	55	Hand numbness, headache	20	2.21	0.83	Dysphagia, hoarseness	Left CPA

No, number; MD, maximum diameter of intracranial arachnoid cyst; Pre, preoperative; Post, postoperative; cm, centimeter; LDVT, lower extremity deep venous thrombosis; CPA, cerebellopontine angle. MD (cm, post) showed the smallest MD of the cyst which was available on MRI or CT image during the follow-up period.

### Imaging examination

All cases in the group underwent CT and/or MRI examinations before and after the operation. In group A, three patients' lesions were in the frontal lobe, three lesions were in the temporal lobe, two lesions were in the cerebellopontine angle (CPA), one lesion was in the saddle, and one lesion was in the prepontine cistern. In group B, two lesions were in CPA, one was in the temporal lobe, and one was in the cerebellum. Because of the irregular shape of the cyst, we measured the maximum diameter of the cyst for comparison. The maximum diameter of each intracranial arachnoid cyst was measured in before and after operation (Table 1).

### Surgical approaches

#### Neuroendoscopic surgery

**Figure 1:**
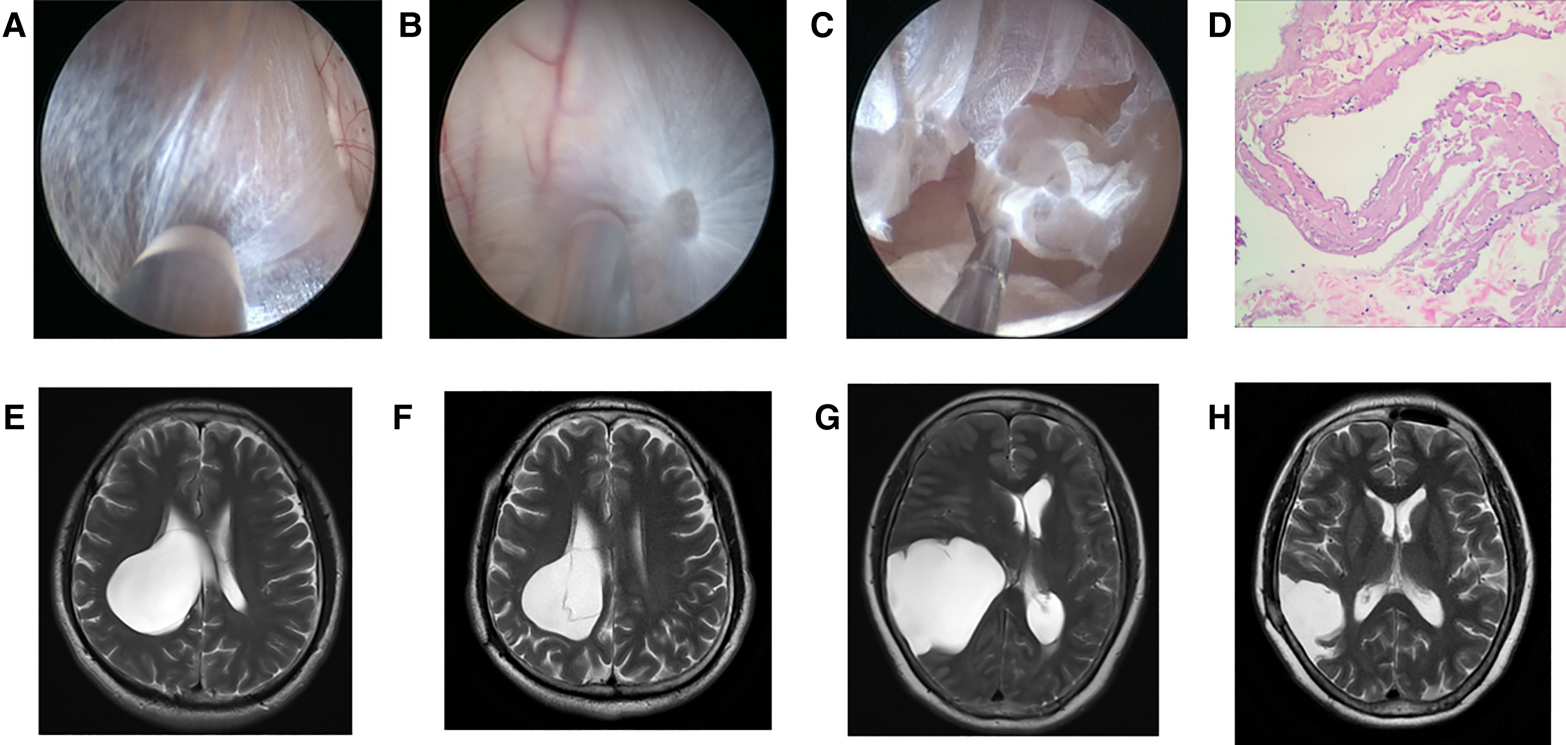
Neuroendoscopic treatment to intracranial arachnoid cyst. (**A**) Lateral wall of intracranial arachnoid cyst before it was incised (No. 6 patient). (**B**) Medial wall of intracranial arachnoid cyst after entering it with neuroendoscope (No. 6 patient). (**C**) Part of medial wall was resected with microshear (No. 6 patient). (**D**) Pathological image of the arachnoid cyst wall which was resected. (**E**) Preoperative T2 MRI image of No. 6 patient. (**F**) T2 MRI image of No. 6 patient at 2 years and 2 months after neuroendoscopic surgery. (**G**) Preoperative T2 MRI image of No. 5 patient. (**H**) T2 MRI image of No. 5 patient at 1 year after neuroendoscopic surgery.

After general anesthesia, one hole was drilled with the use of a bone drill. Then, the dura was suspended and the neuroendoscope was applied. The dura was dissected to expose the arachnoid cyst wall under neuroendoscope. The cyst was cut open, and the cyst fluid was released. In order to keep the cyst from communicating with the cisterna, a cyst-ventricular cisternostomy was performed by bipolar electrocoagulation, and part of the cyst wall was resected with microshear (Figures 1A–C) (12).

### Craniotomy

A craniotomy was performed under general anesthesia to expose the arachnoid cyst, and the parietal arachnoid layer was cut off to release the cystic fluid. The cyst wall was partially or extensively resected under the microscope. The cisternae adjacent to the arachnoid cyst cavity were gently pushed through so that the arachnoid cyst cavity and cisternae could communicate with each other to avoid cyst recurrence after the surgery.

### Follow-up

Patient information was collected from the medical record system of our hospital and followed up by telephone and outpatient clinic (13, 14). Imaging data (CT and MRI) were obtained from the imaging system of our hospital and other communication devices.

### Statistical analysis

Data with normal distribution were described by mean ± standard deviation (*x* ± *s*). The difference in means between the two samples was compared using an independent sample *t*-test. Categorical variables were described by the number of cases and constituent ratio, and the differences between groups were tested by the *χ*^2^ test. A *P* < 0.05 was defined as statistically significant (two tails) (13, 15).

## Results

### Demographic data

A total of nine patients with intracranial arachnoid cyst were included in group A: 3 (33.3%) were male and 6 (66.7%) were female. In group B, there were 2 (50%) male and 2 (50%) female. The patient age was 38.11 ± 5.04 years in group A, and 47.75 ± 7.70 years in group B. The preoperative hospital stay, postoperative in-hospital stay, and total in-hospital stay were not different between the two groups, but the hospital cost of group A was significantly lower than that of group B (47,292.8 vs. 65,151.8 yuan) (Table 2).

**Table 2:** Comparison of clinical data between group A and group B.

Item	Group A (9 cases)	Group B (4 cases)	*P*-value
Age (year, *x* ±* s*)	38.11 ± 5.04	47.75 ± 7.70	0.314
Male/female (cases)	3/6	2/2	0.569
Hospitalization cost (RMB, ten thousand)	4.73 ± 0.47	6.52 ± 0.52	0.045
Total hospital stay (day)	17.11 ± 1.50	13.5 ± 0.87	0.1567
Preoperative hospital stay (day)	4.44 ± 0.55	3.75 ± 0.48	0.460
Postoperative hospital stay (day)	12.67 ± 1.61	9.75 ± 1.11	0.280

### Clinical presentation and follow-up outcomes

**Figure 2:**
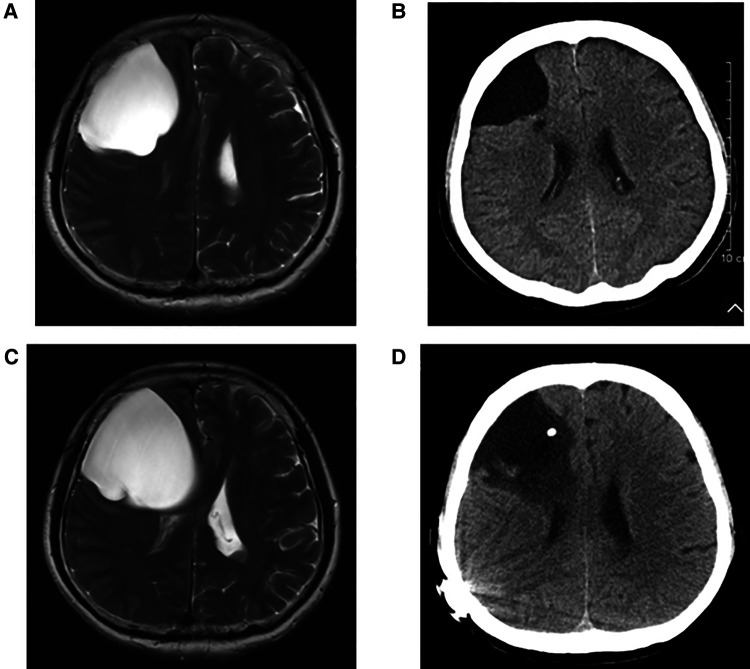
(**A**) Preoperative T2 MRI image of No. 4 patient. (**B**) CT image of No. 4 patient at 1 year and 4 months after neuroendoscopic surgery. (**C**) T2 MRI image of No. 4 patient at 2 years and 6 months after neuroendoscopic surgery. (**D**) CT image of No. 4 patient after intracranial arachnoid cyst-peritoneal shunt.

In group A, four patients presented with dizziness in the preoperative period; three of the four patients had relief of dizziness in the postoperative period. The three patients suffered from headache before surgery, and headache symptoms improved in all of them after surgery. However, two of the three patients had an increase in headache symptoms during the follow-up period (No. 3 and No. 4 patients). Their MRI showed the intracranial arachnoid cysts had grown larger than them in the preoperative period, and they then underwent intracranial arachnoid cyst-peritoneal shunt in our department (No. 4 patient's MRI and CT images are shown in Figure 2). Two patients presented with cerebellar ataxia in the preoperative period, and their symptoms improved after surgery. One patient had epileptic symptoms before surgery, and his frequency of seizures decreased after surgery (Table 3).

**Table 3:** Symptoms and signs in group A and group B.

Symptoms and signs	Group A (9 cases)	Group B (4 cases)
Dizziness	4 (44.4%)	2 (50.0%)
Headache	3 (33.3%)	2 (50.0%)
Ataxia	2 (22.2%)	2 (50.0%)
Seizure	1 (11.1%)	0 (0%)
Dysarthria	1 (11.1%)	0 (0%)
Tinnitus	1 (11.1%)	0 (0%)
Hand tremor	0 (0%)	1 (25.0%)
Hand numbness	0 (0%)	1 (25.0%)

In group B, three patients suffered from headache, and they had relief of headache after surgery. Two patients presented with cerebellar ataxia. After surgery, one patient's symptom was improved, but the other patient presented with symptoms related to the posterior cranial nerve. One patient had aphasia and showed progressive improvement in aphasia during the follow-up period.

**Figure 3:**
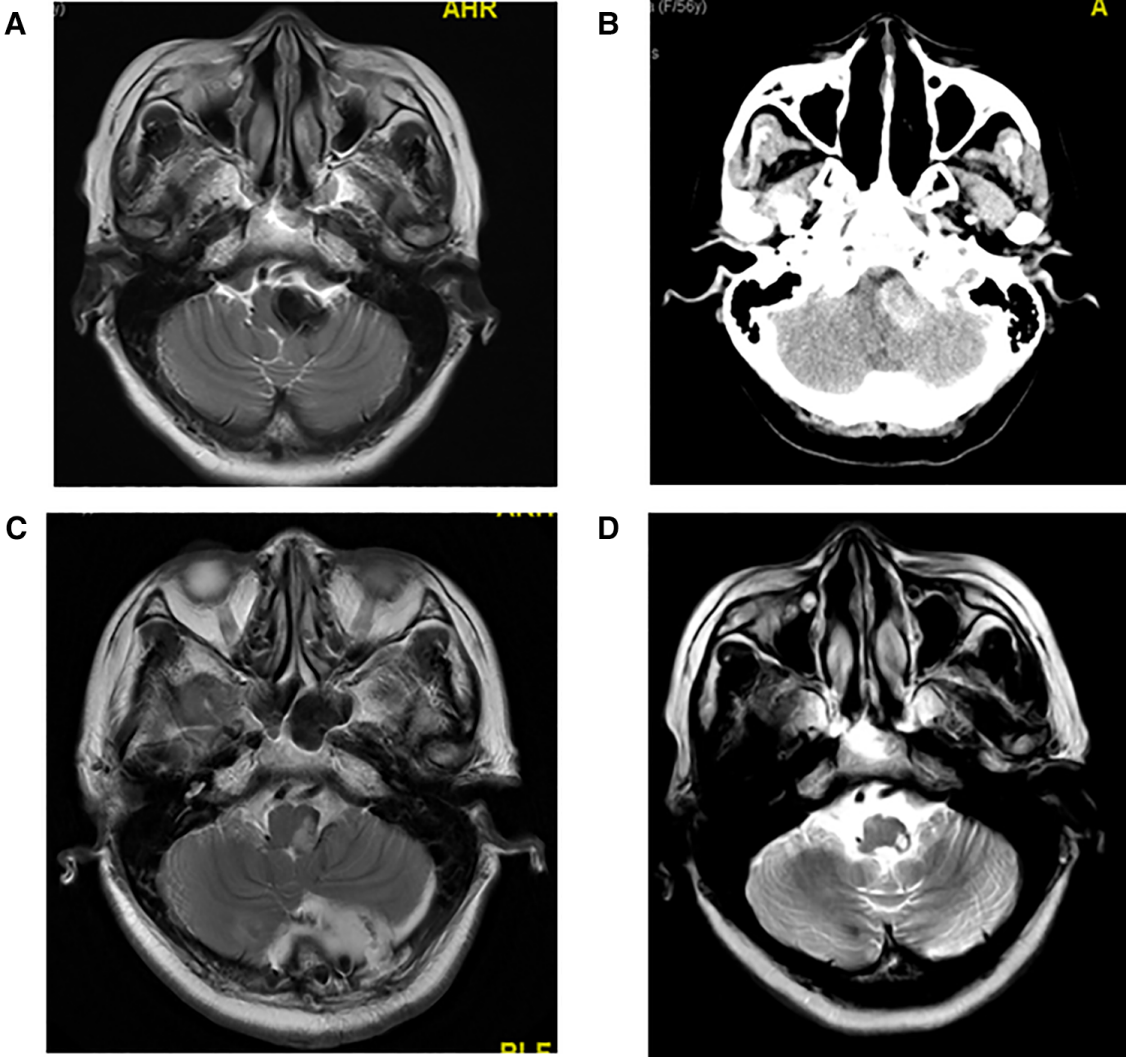
(**A**) Preoperative T2 MRI image of No. 13 patient. (**B**) Preoperative CT image of No. 13 patient with high CT signal. Hemorrhage was observed in the arachnoid cyst during craniotomy. (**C**) T2 MRI image of No. 13 patient at 1 week after craniotomy. (**D**) T2 MRI image of No. 13 patient at 1 year and 10 months after craniotomy.

The maximum diameter of intracranial arachnoid cysts in group A was 6.38 cm on average, and that in group B was 2.97 cm on average, which was significantly different between the two groups (Tables 1, 4). In group A, all of the patients' intracranial arachnoid cysts showed a remarkable reduction in cyst size during the follow-up period (e.g., No. 6 and No. 5 patients' MRI images are shown in Figures 1E–H). However, two patients' cysts recurred, and an intracranial arachnoid cyst-peritoneal shunt was performed. In group B, patients' intracranial arachnoid cysts were resected, then the brain tissue tends to return to the normal anatomical structure (e.g., No. 13 patient's MRI and CT images are shown in Figure 3), and the cyst did not recur during the follow-up period.

**Table 4:** Outcomes in group A and group B.

Item	Group A (9 cases)	Group B (4 cases)	*P*-value
MD (cm, preoperative)	6.38 ± 0.87	2.97 ± 0.66	0.034
MD (cm, postoperative)	5.03 ± 1.07	2.28 ± 0.78	0.138
Postoperative complication	4 (44.44%)	2 (50.0%)	0.852
Follow-up period (month)	34.44 ± 6.90	47 ± 11.14	0.344
Symptom relief (short-term)	9 (100%)	3 (75.0%)	0.119
Symptom relief (long-term)	7 (77.78%)	3 (75.0%)	0.913

MD, maximum diameter of intracranial arachnoid cyst; cm, centimeter.

## Discussion

Intracranial arachnoid cysts are common intracranial space-occupying lesions. Previous studies reported the incidence of this cyst in males is the same as with that in females (16). Intracranial arachnoid cysts are often found in the middle cranial fossa, posterior cranial fossa, suprasellar, and quadrigeminal bodies (7, 9). Some intracranial arachnoid cyst patients present with clinical symptoms, such as headache, nausea, vomiting, dizziness, seizures, and cranial hypertension (7, 8). Although it is still controversial about the operation's indication to intracranial arachnoid cysts, patients with clinical symptoms are suggested to surgical therapy.

In this study, we demonstrated that short-term outcomes for patients who underwent endoscopic surgery in group A were the same as those in group B, and that hospital costs were significantly lower in group A than in group B. However, in group A patient No. 3, the intracranial arachnoid cysts recurred after neuroendoscopic surgery a year later, and she underwent intracranial arachnoid cyst-peritoneal shunt. No. 4 patient in group A resuffered from dizziness in 2.5 years after neuroendoscopic surgery and presented with intracranial arachnoid cyst recurrence in the right frontal lobe, and she also underwent an intracranial arachnoid cyst-peritoneal shunt. The recurrence rate in group A is 22.2% but in group B is 0%; thus, long-term outcome in group B might be better than that in group A. After neuroendoscopic surgery, the cyst became smaller, and the defect of the arachnoid cyst may regrow and fuse, then cyst-cisterna communication was interrupted. This might be the reason for the recurrence.

Neuroendoscopic surgery is a minimally invasive technique that allows for direct visualization of intracranial area. Few previous studies compared the length of hospital stay and hospitalization costs of these two surgical methods for treating adult intracranial arachnoid cysts. In the current study, the total in-hospital stay was 17.1 days vs. 13.5 days (group A vs. group B), but the hospital cost of group A was significantly lower than that of group B (47,292.8 vs. 65,151.8 yuan). This study compared the economic burden and found that neuroendoscopic surgery could reduce hospitalization costs and the economic burden on patients.

**Figure 4:**
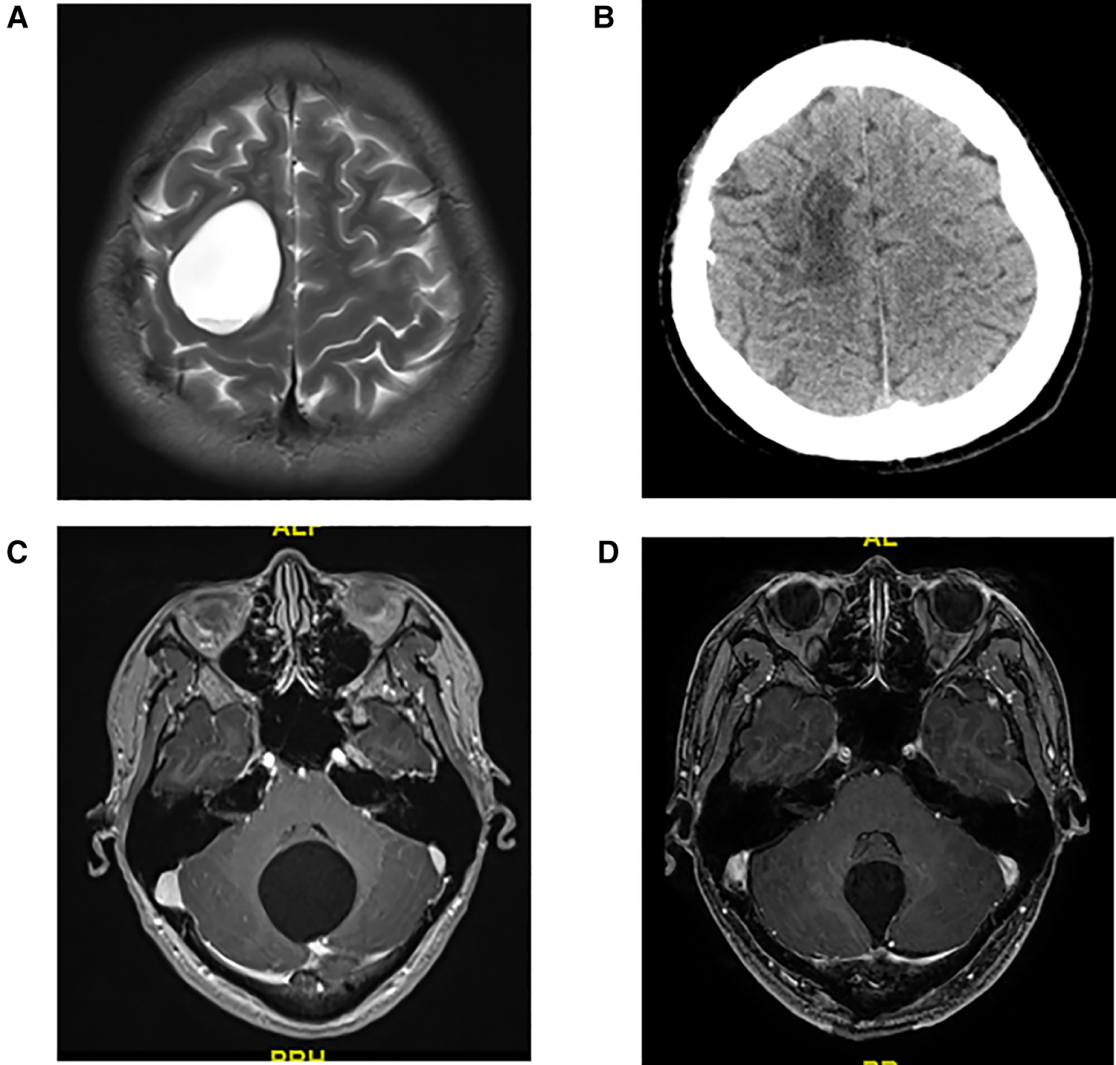
(**A**) Preoperative T2 MRI image of No. 7 patient. (**B**) CT image of No. 7 patient at 1 month after neuroendoscopic surgery. (**C**) Preoperative T1 MRI image of No. 11 patient. (**D**) T1 MRI image of No. 11 patient at 1 week after craniotomy.

It is still controversial which surgical approach is suitable for adult intracranial arachnoid cysts patients (9, 11). In the current study, we did not use the volume to compare the intracranial arachnoid cyst because in some cases the lesion shape was irregular and the area could not be calculated; thus, we used the maximum diameter of the lesion to compare the lesions. The maximum diameter of intracranial arachnoid cysts in group A is significantly larger than in group B. A large intracranial arachnoid cysts may have a wide range of adhesion with normal brain tissue, and resection might cause more serious postoperative complications; thus, in our study, neuroendoscopic surgery was applied to patient with larger intracranial arachnoid cysts (maximum diameter > 4.5 cm), and craniotomy was used to treat patient with small intracranial arachnoid cysts (maximum diameter < 3.0 cm). The selection of the surgical approach to the cyst with maximum diameter between 3.0 and 4.5 cm, depending on the cyst's the position, was close to the function area. For example, patient No. 7 had the lesion in motor function area, and neuroendoscopic surgery was performed to avoid motor function impairment (Figures 4A,B). No. 11 patient had the cyst in the dorsal part of the cerebellum, and craniotomy was used to complete resection of the lesion (Figures 4C,D).

Intracranial arachnoid cyst in an adult patient with surgical indication is an extremely rare case. Due to low incidence rate, this study collected only 14 cases, which may have an impact on the results of the study, and we plan to recruit more patients for a further study. In addition, prospective studies are needed to provide strong evidence, that can provide a basis for the choice of surgical methods.

## Conclusion

Both neuroendoscopic and craniotomy have good curative effects for the treatment of intracranial arachnoid cysts. There was no significant difference in the outcomes between the two surgical techniques. The cost of hospitalization can be reduced with neuroendoscopic surgery. Neuroendoscopic treatment is recommended for large intracranial arachnoid cysts (maximum diameter > 4.5 cm), and craniotomy is suitable to small intracranial arachnoid cysts (maximum diameter < 3.0 cm).

## Data Availability

The original contributions presented in the study are included in the article/Supplementary Material, further inquiries can be directed to the corresponding author/s.
