# Corrigendum: Effect comparison of neuroendoscopic vs. craniotomy in the treatment of adult intracranial arachnoid cyst

**DOI:** 10.3389/fsurg.2023.1167009

**Published:** 2023-05-03

**Authors:** Jianfeng Liang, Kai Li, Bin Luo, Jun Zhang, Peng Zhao, Changyu Lu

**Affiliations:** ^1^ Department of Neurosurgery, Peking University International Hospital, Beijing, China; ^2^ Department of Neurosurgery, Beijing Tiantan Hospital, Capital Medical University, Beijing, China

**Keywords:** neuroendoscopic, craniotomy, intracranial arachnoid cyst, surgery, complication

A Corrigendum on Effect comparison of neuroendoscopic vs. craniotomy in the treatment of adult intracranial arachnoid cyst By Liang J, Li K, Luo B, Zhang J, Zhao P and Lu C. (2023) Front. Surg. 10:1054416. doi: 10.3389/fsurg.2022.1054416

**Incorrect Affiliation**

In the published article, there was an error in affiliation [1]. Instead of “[Department of Neurosurgery, International Hospital, Peking University, Beijing, China]”, it should be “[Department of Neurosurgery, Peking University International Hospital, Beijing, China]”.

The authors apologize for this error and state that this does not change the scientific conclusions of the article in any way. The original article has been updated.

